# Delayed functional expression of neuronal chemokine receptors following focal nerve demyelination in the rat: a mechanism for the development of chronic sensitization of peripheral nociceptors

**DOI:** 10.1186/1744-8069-3-38

**Published:** 2007-12-12

**Authors:** Sonia Bhangoo, Dongjun Ren, Richard J Miller, Kenneth J Henry, Jayana Lineswala, Chafiq Hamdouchi, Baolin Li, Patrick E Monahan, David M Chan, Matthew S Ripsch, Fletcher A White

**Affiliations:** 1 Molecular Pharmacology and Structural Biochemistry, Northwestern University, Chicago, IL, USA; 2 Eli Lilly and Company, Lilly Corporate Center, Indianapolis, IN, USA; 3 Cell Biology, Neurobiology & Anatomy, Loyola University – Chicago, Maywood, IL, USA; 4 Anesthesiology, Loyola University – Chicago, Maywood, IL, USA

## Abstract

**Background:**

Animal and clinical studies have revealed that focal peripheral nerve axon demyelination is accompanied by nociceptive pain behavior. C-C and C-X-C chemokines and their receptors have been strongly implicated in demyelinating polyneuropathies and persistent pain syndromes. Herein, we studied the degree to which chronic nociceptive pain behavior is correlated with the neuronal expression of chemokines and their receptors following unilateral lysophosphatidylcholine (LPC)-induced focal demyelination of the sciatic nerve in rats.

**Results:**

Focal nerve demyelination increased behavioral reflex responsiveness to mechanical stimuli between postoperative day (POD) 3 and POD28 in both the hindpaw ipsilateral and contralateral to the nerve injury. This behavior was accompanied by a bilateral increase in the numbers of primary sensory neurons expressing the chemokine receptors CCR2, CCR5, and CXCR4 by POD14, with no change in the pattern of CXCR3 expression. Significant increases in the numbers of neurons expressing the chemokines monocyte chemoattractant protein-1 (MCP-1/CCL2), Regulated on Activation, Normal T Expressed and Secreted (RANTES/CCL5) and interferon γ-inducing protein-10 (IP-10/CXCL10) were also evident following nerve injury, although neuronal expression pattern of stromal cell derived factor-1α (SDF1/CXCL12) did not change. Functional studies demonstrated that acutely dissociated sensory neurons derived from LPC-injured animals responded with increased [Ca^2+^]_i _following exposure to MCP-1, IP-10, SDF1 and RANTES on POD 14 and 28, but these responses were largely absent by POD35. On days 14 and 28, rats received either saline or a CCR2 receptor antagonist isomer (CCR2 RA-**[R]**) or its inactive enantiomer (CCR2 RA-**[S]**) by intraperitoneal (i.p.) injection. CCR2 RA-[**R**] treatment of nerve-injured rats produced stereospecific bilateral reversal of tactile hyperalgesia.

**Conclusion:**

These results suggest that the presence of chemokine signaling by both injured and adjacent, uninjured sensory neurons is correlated with the maintenance phase of a persistent pain state, suggesting that chemokine receptor antagonists may be an important therapeutic intervention for chronic pain.

## Introduction

Inflammatory events induced by nerve injury are thought to play a central role in the pathogenesis of inflammatory pain. The production and release of molecules that mediate the acute inflammatory response include bradykinin, tachykinins, serotonin, histamine, ATP and cytokines such as tumor necrosis factor-alpha (TNFα), interleukin 1-β (IL-1β), and interleukin-6 (IL-6). Many of these molecules, which are produced in association with acute inflammatory responses, are known to induce hyperalgesia [1,2]

Chemokines, which also contribute to the development of inflammatory pain states, can directly excite subsets of sensory neurons [3-8]. This excitation is likely to be due to transactivation of ion channels, such as TRPV1 and TRPA1, expressed by sensory nerves [9,10]. As such, it is quite possible that a prolonged *de novo *expression of chemokines and/or their cognate receptors by sensory neurons following peripheral nerve injury may be central to the development and/or maintenance of chronic pain states. Indeed, we previously demonstrated that in a rodent model of spinal stenosis, chronic compression of the DRG (CCD), produced a delayed but chronic expression of both the chemokine receptor CCR2 and its ligand, the chemokine MCP-1/CCL2 in lumbar DRGs [8]. Furthermore, MCP-1/CCL2 depolarized or increased the excitability of several subpopulations of sensory neurons, including nociceptors, in both the intact and dissociated DRG [6,8]. Interestingly, mice deficient in the chemokine receptor, CCR2, exhibit an impaired neuropathic pain response following partial nerve ligation [11].

In order to fully understand the extent and significance of neuronal chemokine signaling in states of pain hypersensitivity, we examined whether induction of a focal demyelination of the sciatic nerve, a known rodent model of neuropathic pain [12], produced changes in the neuronal expression of certain key chemokines previously shown to be extensively upregulated in peripheral neuroinflammatory responses [3,13-16]. These chemokines included monocyte chemoattractant protein-1 (MCP-1/CCL2), interferon γ-inducing protein-10 (IP-10/CXCL10), regulated on activation normal T cell expressed and released (RANTES/CCL5) and stromal cell derived factor-1 (SDF1/CXCL12) and their cognate receptors (CCR2, CXCR3, CCR5 and CXCR4, respectively).

We now demonstrate that focal peripheral nerve demyelination in the right thigh of the rat produces chronic bilateral nociceptive behavior as measured by hindpaw withdrawal. Together with the ongoing display of nociceptive behavior is a delayed upregulation of several C-C and C-X-C chemokines and their cognate receptors by sensory neurons. Though there is an initial delay in ligand/receptor upregulation, the continued expression of neuronal chemokine/receptors appears to correlate with changes in chronic nociceptive behavior. Furthermore, administration of a CCR2 receptor antagonist produced an attenuation of the nociceptive behavior, further highlighting the potential role of chemokine signaling in states of neuropathic pain.

Parts of this study have been previously published in abstract form [17,18].

## Methods

### Animals

Pathogen-free, adult female Sprague-Dawley rats (150–200 g; Harlan Laboratories, Madison, WI) were housed in temperature (23 ± 3°C) and light (12-h light:12-h dark cycle; lights on at 07:00 h) controlled rooms with standard rodent chow and water available ad libitum. Experiments were performed during the light cycle. Animals were randomly assigned to the treatment groups. These experiments were approved by the Institutional Animal Care and Use Committee of Loyola University, Chicago. All procedures were conducted in accordance with the Guide for Care and Use of Laboratory Animals published by the National Institutes of Health and the ethical guidelines of the International Association for the Study of Pain. All animals were randomly assigned to either treatment or control groups.

### Sciatic nerve demyelination

Animals were anesthetized with 4% isoflurane and maintained on 2% isoflurane (Halocarbon, River Edge, NJ) in O_2_. For all demyelination experiments, lysophosphatidylcholine (LPC), (type V, 99% pure; Sigma-Aldrich, St. Louis, MO) was dissolved in buffered sterile saline (pH 7.2) to give a final concentration of 10 mg/ml. The right sciatic nerve of the rat was exposed at the mid-thigh level under sterile conditions. A sterile polyvinyl acetal (PVAc) sponge (Ivalon, San Diego, CA), 2-mm × 2-mm soaked in 7 μl of LPC, was placed adjacent to the sciatic nerve. The dermal incision site was closed with 5.0 suture thread. Sham control animals were prepared as described above, but buffered sterile saline was used in place of LPC plus saline. Some control rats were also given an intramuscular injection of LPC (10 ul, 1%) into the gastrocnemius muscle.

### Drugs and method of administration

A CCR2 receptor antagonist and its inactive enantiomer were employed in this study [19]. The CCR2 antagonist active enantiomer's full name is (R)-4-Acetyl-1-(4-chloro-2-fluorophenyl)-5-cyclohexyl-3-hydroxy-1,5-dihydro-2H-pyrrol-2-one (CCR2 RA **[R]**). The inactive enantiomer is (S)-4-Acetyl-1-(4-chloro-2-fluorophenyl)-5-cyclohexyl-3-hydroxy-1,5-dihydro-2H-pyrrol-2-one (CCR2 RA **[S]**) (Additional file 1). Both were employed as Na+ salts. The affinity of CCR2 RA **[R] **for the rat CCR2 receptor is > 4000 that of the S-isomer. Both compounds were freshly prepared in saline on the day of the experiment (10 mg/kg). Active and inactive enantiomer and vehicle-treated groups (n = 8 per group) were given a one-time intraperitoneal (i.p.) injection one hour prior to behavioral testing.

### Foot withdrawal to punctate mechanical indentation

The incidence of foot withdrawal was measured in response to mechanical indentation of the plantar surface of each hind paw with sharp, Von Frey-type nylon filaments. Mechanical stimuli were applied with seven filaments, each differing in the bending force delivered (10, 20, 40, 60, 80, 100, and 120 mN), but each fitted with the same metal cylinder with a flat tip and a fixed diameter of 0.2 mm [3]. In each behavioral testing sequence, the operator was blinded to the animal treatment condition.

The rat was placed on a metal mesh floor and covered with a transparent plastic dome. Typically, the animals rest quietly in this situation after an initial few minutes of exploration. Animals were habituated to this testing apparatus for 15 minutes a day, two days prior to the behavioral assays. Following acclimation, each filament was applied to six spots spaced across the hind paw. The filaments were tested in order of ascending force, with each filament delivered in sequence from the 1^st ^to the 6^th ^spot alternating from one hind paw to the other. The duration of each stimulus was 1 second and the interstimulus interval was 10–15 seconds. A cutoff value of 120 mN was used; animals that did not respond at 120 mN were assigned that value [3,20].

The incidence of foot withdrawal was expressed as a percentage of the six applications of each filament as a function of force. A Hill equation was fitted to the function (Origin version 6.0, Microcal Software, Northhampton MA) relating the percentage of indentations eliciting a withdrawal to the force of indentation. From this equation, the paw withdrawal threshold (PWT) force was obtained and defined as the force corresponding to a 50% withdrawal. At least a -20 mN difference from the baseline PWT in a given animal is representative of neuropathic pain [3].

Measurements were taken on three successive days before surgery. Postoperative testing was performed on one, three and seven days after surgery and weekly thereafter for the duration of the experiment. PWT values were statistically analyzed for each foot separately and for the significance of differences between the average of the three preoperative tests and the mean obtained for each postoperative test. The same statistical analyses are applied to the slopes of the logistic functions from which the PWTs are derived. The experimenter was blinded to both the injury condition of the animal and the drugs utilized in all behavioral trials.

### Foot withdrawal to thermal stimulus

To evaluate the PWT to thermal stimulation, we used the Hargreaves' plantar test apparatus (Ugo Basile, Varese, Italy). Rats were placed on a 2-mm-thick glass floor; a mobile infrared heat generator with an aperture of 10 mm was aimed at the rat's hind paw under the floor. Following activation of the heat source, the reaction time (the withdrawal latency of the hindpaw) of the rat was recorded automatically. A shortening of the withdrawal latency indicated thermal hyperalgesia. The temperature of the glass floor was kept at 22.5–23.5°C. Measurements of the withdrawal latency of the paw began after the rats were habituated to the testing environment (IR setting = 70). The measurements were repeated four times, at 5 min intervals, on each paw, and the initial pair of measurements was not used. The averages of the three remaining pairs of measurements taken were employed as data.

### In situ hybridization

In situ hybridization histochemistry for chemokine receptors was performed by using digoxigenin-labeled riboprobes. Adult female Sprague-Dawley rats were euthanized using carbon dioxide. L_4_L_5 _DRGs ipsi- and contralateral to LPC nerve injury were rapidly removed, embedded in OCT compound (Tissue Tek, Ted Pella, Inc., Redding, CA) and frozen. Sections were serially cut at 14 μm. The CCR2 probe was prepared as described [8]. Briefly, an 848-bp CCR2 cDNA fragment (nucleotides 489–1336 of GenBank no. U77349) was cloned by PCR using rat spleen cDNA. The resulting PCR product was subcloned into a pGEM-T Easy vector and sequenced to ensure identity for riboprobe use. The CCR2 template was linearized with SacII to generate a probe of 950 bases by using SP6 polymerase. Signals were visualized by using NBT/BCIP reagents (Roche Diagnostics/Boehringer Mannheim, Indianapolis, IN) in the dark for 2–20 h depending upon the abundance of the RNA. Images were captured using brightfield or differential interference contrast optics with a Nikon E600 fluorescent microscope (NikonUSA, Melville, NY) fitted with a charge-coupled device camera (Retiga EXi, Q-Imaging Corporation, Vancouver, BC). CCR2 mRNA expression studies were used for receptor localization because of the failure of immunocytochemistry to detect neuronal CCR2 protein.

The RANTES plasmid was a gift from Dr. Richard M. Ransohoff (Cleveland Clinic Foundation). The RANTES plasmid was sub-cloned into a pGEM vector. The plasmid templates were linearized with restriction enzyme digestion.

The CXCR4 and SDF-1 probes were generated as described previously [21]. For the CXCR3 and CCR5 probes, we used the CD1 mouse brain cDNA. The CXCR3 cDNA fragment was amplified using the forward primer 5'-gag gtt agt gaa cgt caa gtg-3' and the reverse primer 5'-tgg aga cca gca gaa cag cta g-3'. The CCR5 fragment used the forward primer 5'-tgg att atg gta tgt cag cac cc-3'and the reverse primer 5'-tcg att atg gta tgt cag cac cc-3'. All PCR fragments were subcloned into a pCR II-TOPO vector, and were verified by restriction analysis and automated DNA sequencing (Perkin Elmer, Boston MA)

The plasmid templates were linearized by restriction enzyme digestion. Then transcription was labeled by digoixigenin (Roche Applied Science, Indianapolis, IN).

### Immunocytochemical labeling

Adult female Sprague-Dawley rats were deeply anesthetized with isoflurane and transcardially perfused with saline followed by 4% paraformaldehyde. Lumbar ganglia associated with the sciatic nerve ipsilateral and contralateral to focal nerve demyelination injury (n = 6) or sham treatment (n = 6) were immediately removed following behavior on POD 7 or 14 and postfixed for 4 hours. Additional lumbar DRGs were removed from naïve, behaviorally tested rats (n = 6). Lumbar DRGs were encoded at the outset and processed in random order. Sagittal sections of the DRG were serially cut at 14 μm onto SuperFrost microscope slides (Fisher Scientific, Pittsburgh PA). At least 6 sections were obtained for immunocytological analysis per DRG. Tissue was processed such that DRG sections on each slide were at intervals of 80 um. Slides were incubated with blocking buffer (3% BSA/3% horse serum/0.4% Triton-X; Fisher Scientific, Pittsburgh PA) for 1 hour, followed by overnight incubation with the rabbit polyclonal antisera generated against MCP-1 (1:500; Chemicon, Temecula, CA), IP-10 (1:1000, Abcam, Cambridge MA) or CCR2 (1:500; Aviva Systems Biology, San Diego CA) at room temperature. After primary incubation, secondary antibodies (anti-rabbit conjugated to CY3, made in donkey at 1:800; Jackson ImmunoResearch, West Grove, PA) were used to visualize cells. Some experiments were augmented with the addition of *Griffoniasimplicifolia *I-isolectin B4 (IB4) conjugated with fluorescein (1 mg/1 ml; Sigma, St. Louis MO). Slides were washed in PBS for 5 min each (×3) and coverslipped with a PBS/glycerol solution. All tissue sections were also stained with Hoechst 33258 nuclear marker (Invitrogen Corporation, Carlsbad CA).

Tissue sections were analyzed for the presence of IB_4_-binding neurons and either MCP-1, IP-10 or CCR2. Because a stereological approach was not employed in this study, quantification of the data may represent a biased estimate of the actual numbers of immunopositive neurons. The proportions of immunoreactive neurons were determined from the total number of Hoescht-positive neuronal nuclei present in a tissue section. The overall diameter and brightness of the Hoescht-positive neuronal nuclei allowed for a clear delineation between neurons and non-neuronal cells in the DRG. At least 5000 neuronal profiles from six animals (minimum of 625 cells per ganglia) were quantified for each cell type in the single neuronal marker study and for each combination of cellular markers. Quantification of cell numbers, degree of colocalization and cell diameters was determined using ImagePro Plus (Media Cybernetics, Silver Spring, MD). As noted above, individuals conducting cell quantification were blinded to the treatment conditions. Data are represented as means ± SEM%.

### Preparation of acutely dissociated dorsal root ganglion neurons

The L_4_–L_5 _DRG were acutely dissociated using methods described by Ma and LaMotte [22]. Briefly, L_4 _and L_5 _DRG were removed from control or LPC-treated animals at various post-operative day timepoints. The DRGs were treated with collagenase A and collagenase D in HBSS for 20 minutes (1 mg/ml; Roche Applied Science, Indianapolis, IN), followed by treatment with papain (30 units/ml, Worthington Biochemical, Lakewood, NJ) in HBSS containing .5 mM EDTA and cysteine at 35°C. The cells were then dissociated via mechanical trituration in culture media containing 1 mg/ml bovine serum albumin and trypsin inhibitor (1 mg/ml, Sigma, St. Louis MO). The culture media was Ham's F12 mixture, supplemented with 10% fetal bovine serum, penicillin and streptomycin (100 ug/ml and 100 U/ml) and N2 (Life Technologies). The cells were then plated on coverslips coated with poly-L-lysine and laminin (1 mg/ml) and incubated for 2 hours before more culture media was added to the wells. The cells were then allowed to sit undisturbed for 12–15 hours to adhere at 37°C (with 5% CO_2_).

### Intracellular Ca^2+ ^imaging

The dissociated DRG cells were loaded with fura-2 AM (3 uM, Molecular Probes/Invitrogen Corporation, Carlsbad CA) for 25 minutes at room temperature in a balanced salt solution (BSS) [NaCl (140 mM), Hepes (10 mM), CaCl_2 _(2 mM), MgCl_2 _(1 mM), Glucose (10 mM), KCl (50 mM)]. The cells were rinsed with the BSS and mounted onto a chamber that was placed onto the inverted microscope and continuously perfused with BSS at a rate of 1 ml/min. Intracellular calcium was measured by digital video microfluorometry with an intensified CCD camera coupled to a microscope and MetaFluor software (Molecular Devices Corporation, Downington, PA). Cells were illuminated with a 150 W xenon arc lamp, and the excitation wavelengths of the fura-2 (340/380 nm) were selected by a filter changer. Chemokines were applied directly into the coverslip bathing solution after the perfusion was stopped. If no response was seen within 1 minute, the chemokine was washed out. For all experiments, MCP-1, SDF1, RANTES and IP10 were added to the cells in random order, after which capsaicin, high K+ (50 K) and ATP were added. The chemokines used were purchased from R & D Systems (Minneapolis, MN), and all were used at a concentration of 100 nm to ensure maximal activation. They were reconstituted in 0.1%BSA/PBS, and aliquots were stored at -20°C.

### Statistical Analyses

Data is presented as the mean ± SEM, unless otherwise noted. GB-Stat School Pack software (Dynamic Microsystems, Inc. Silver Springs, MD) was used to statistically evaluate all data. The significance difference was determined by two-way ANOVA with Bonferroni's post-hoc test for animal behavior. The one way ANOVA with a Dunnett's Multiple Comparison test was used to analyze the differences between naïve, sham and experimental groups. A difference of p < 0.05 was considered significant.

## Results

### Mechanical stimuli elicit bilateral tactile hyperalgesia following LPC-induced sciatic nerve demyelination

**Figure 1:**
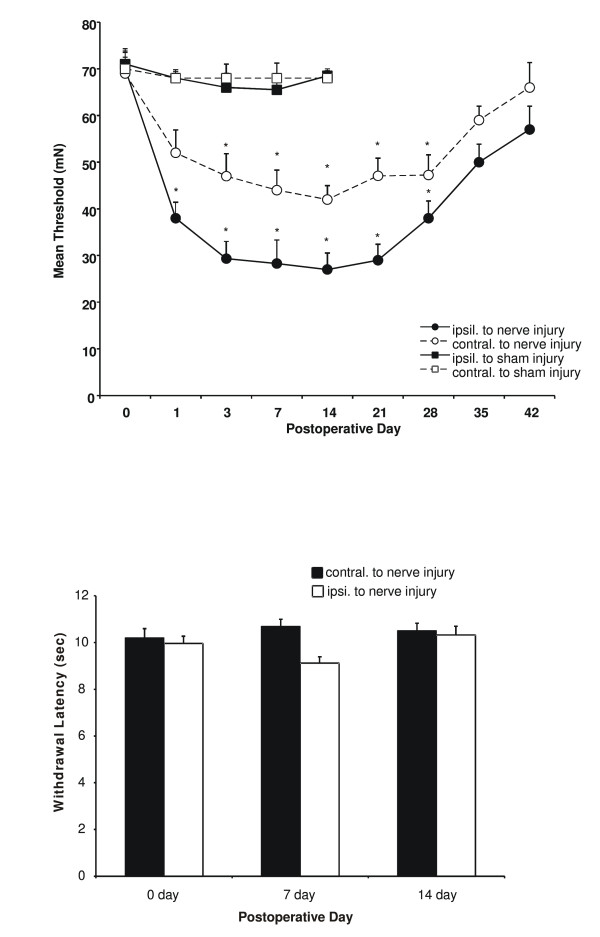
Mean threshold force required for paw withdrawal to Von Frey stimulation at 1, 3, 7, 14, 21, 28, 35 and 42 days following LPC-induced focal nerve demyelination. Each data point is the mean threshold (± SE) force on the hindpaw ipsilateral (black circle) or contralateral (white circle) to the focal nerve injury site eliciting a withdrawal response (n = 10). Reduced behavioral thresholds for the hindpaw ipsilateral to the nerve lesion were significantly different from pre-operative baseline on postoperative days 1–28. The threshold force for the hindpaw contralateral to the nerve lesion did not reach significance until postoperative day 3, and significant differences were observed until postoperative day 28. The time course of sham injury (n = 6) is also represented but did not differ from the uninjured animals. Analysis was performed using two-way ANOVA followed by the Bonferroni post-hoc pair-wise comparisons (*p < 0.01).**B) **LPC-induced focal nerve demyelination did not produce changes in thermal responses as assessed by the Hargreaves test. Each bar is the mean withdrawal latency (± SE) of the hindpaw ipsilateral (white bar) or contralateral (black bar) to the focal nerve demyelination injury at postoperative day 7 and 14 (n = 10).

To study changes in behavioral sensitivity following LPC-induced nerve demyelination, we investigated alterations in the paw withdrawal threshold (PWT) force of indentation (produced by von Frey filaments) necessary for eliciting a flexion hindpaw withdrawal reflex. At POD1, the PWT ipsilateral to the LPC-induced sciatic nerve demyelination was significantly reduced when compared to pre-surgical PWTs (Fig. 1A). The force required to elicit a paw withdrawal steadily declined until POD14, before gradually returning to near pre-surgical levels by POD35. These PWTs met the pre-determined levels indicative of hyperalgesia (-20 mN force) between POD1 and POD28. Changes in behavior were also observed in the hind paw contralateral to the LPC-induced nerve injury (Fig. 1A). These behavioral changes met the pre-determined PWT levels indicative of hyperalgesia between POD3 and POD28 (Fig. 1A). Vehicle-treated sham operated rodents did not exhibit a PWT decrease that was significant at any time point up to POD14. Animals given intramuscular injections of LPC into the gastrocnemius muscle (10 ul, 1% LPC) did not develop cutaneous hyperalgesia (n = 3, data not shown). These data indicate that a unilateral LPC-induced nerve demyelination results in bilateral tactile hyperalgesia.

### Effect of unilateral focal nerve demyelination on thermal thresholds

In contrast to the effectiveness of LPC-induced focal nerve demyelination in altering mechanical PWTs, focal nerve demyelination had little if any effect on thermal responsivity from POD0 to POD14 (Fig. 1B).

### CCR2, CXCR4, CXCR3 and CCR5 upregulation in L_4_–L_5 _DRG after unilateral LPC-induced nerve demyelination

**Figure 2:**
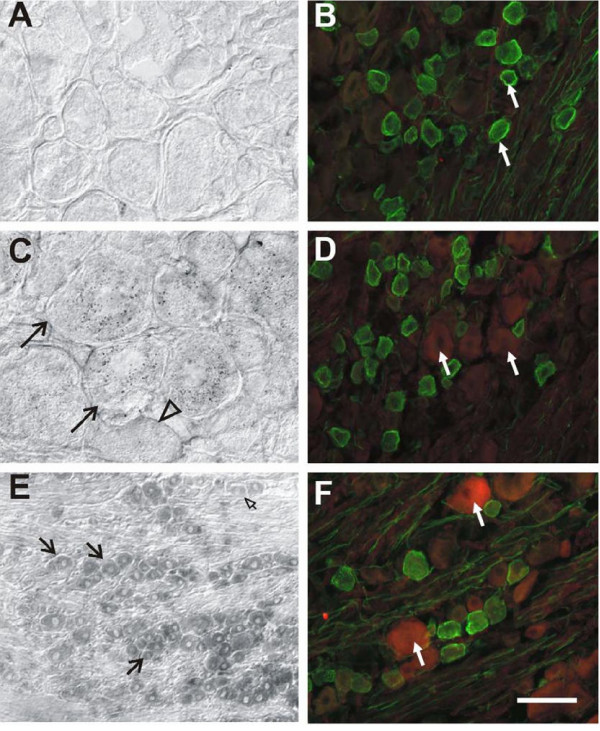
Expression of CCR2 mRNA and protein immunoreactivity in rat lumbar DRG ipsilateral to focal nerve demyelination. **A**) Lumbar DRG removed from vehicle-treated animals at POD7 did not exhibit CCR2 mRNA expression (n = 5). **B**) Many lumbar DRG neurons in vehicle-treated rats sensory neurons were positive for isolectin IB_4_, a neuronal phenotype that distinguishes some C-fiber nociceptors (green cells). There was no evidence of CCR2 protein expression in sham animals (n = 5). **C**) Lumbar DRG neurons from nerve-injured rats on POD7 exhibited CCR2 mRNA transcripts in some small and medium diameter neurons (black arrows). Open black arrowhead indicates a neuron without CCR2 mRNA transcripts (n = 4). **D**) Lumbar DRG neurons from a rat subjected to focal nerve demyelination exhibited few CCR2 immunopositive (white arrows) sensory neurons (n = 4). **E**) Many lumbar DRG neurons on POD14 exhibited CCR2 mRNA transcripts (black arrows). Open arrowhead indicates non-labeled neuron. **F**) CCR2 immunoreactivity was present in an increased number of neurons at POD14 (white arrows; n = 5). Scale bar is; 30 μm (**A**, **C**), 50 μm (**B**, **D**, **F**), and 100 μm (**E**).

**Figure 3:**
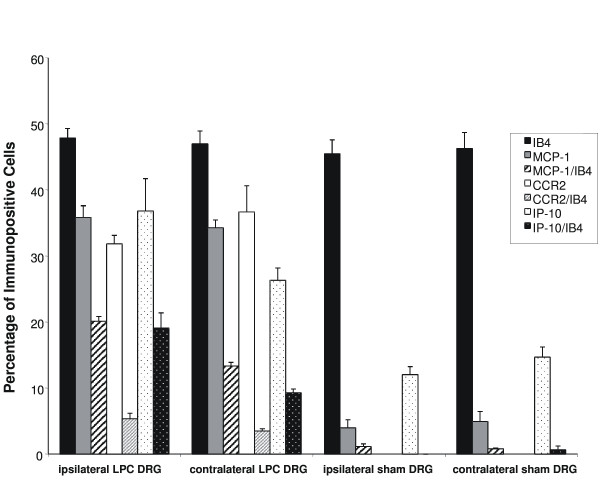
Percentage of MCP-1, CCR2 and IP-10 immunoreactive neurons with IB_4_-positive neuronal profiles on POD 14. MCP-1 expression was increased by LPC-induced nerve injury within the IB_4_-labeled neuronal group in both the DRG ipsilateral and contralateral to the nerve injury. Sham-injury treatment did not produce significant changes in the extent of MCP-1/IB_4 _colocalization. CCR2 expression was increased by LPC-induced nerve injury within IB_4_-labeled neuronal group in both the DRG ipsilateral and contralateral to the nerve injury. Like, MCP-1, sham-injury treatment did not produce significant changes in CCR2/IB4 colocalization in either DRG ipsi- or contralateral to the sham injury. IP-10 expression was increased by LPC-induced nerve injury within IB_4_-labelled neuronal group in both the DRG ipsilateral and contralateral to the nerve injury, while sham-injury treatment did not produce significant changes in IP-10/IB_4 _colocalization. Comparisons of immunoreactive cell percentages were made between LPC-treatment and sham-treated animals. Data represent means ± SE. Analysis was performed using two-way ANOVA followed by the Bonferroni post-hoc pair-wise comparisons (*p < 0.01).

We previously reported upregulation of CCR2 chemokine receptor signaling in association with chronic compression of the DRG [8]. We therefore examined the state of CCR2 expression in association with LPC-induced demyelination. L_4_L_5 _DRGs removed from naïve (data not shown) and vehicle-treated rats at POD7 did not express CCR2 mRNA (Fig. 2A) or CCR2 immunoreactivity (Fig. 2B). Small and medium diameter L_4_L_5 _neurons ipsilateral to the focal nerve lesion exhibited low levels of both CCR2 mRNA transcripts at POD7 (Fig. 2C) and CCR2 immunoreactivity (Fig. 2D). By POD14, many sensory neurons of all diameters exhibited CCR2 mRNA in L_4_L_5 _DRGs both ipsilateral and contralateral to LPC-induced injury (Figs. 2E, 3). Immunoreactivity for neuronal CCR2 at POD14 (Fig. 2F) was also increased relative to POD7. Neuronal binding of the plant isolectin, *Griffoniasimplicifolia *B_4 _(IB_4_) in the rat DRG distinguishes a population of C-fiber nociceptors [23,24]. We double-labeled the sections stained for CCR2 protein with IB_4_. Many IB_4_-binding neurons were present in lumbar DRG of naïve, sham-operated rats and those subjected to LPC-induced focal nerve demyelination (Fig. 2B, D, F). These neurons displayed strong labeling of the plasma membrane, as well as perinuclear staining that in all likelihood represents the Golgi apparatus [25]. Quantitative analysis of neurons positive for CCR2 revealed that 33.35 ± 2.16% of sensory neurons ipsilateral and 35.87 ± 3.36% contralateral to the nerve lesion were positive for the chemokine receptor. Relatively few (<6%) CCR2-positive neurons were colocalized with IB_4_-binding neurons ipsilateral (Fig. 2F and Fig. 3) or contralateral to the nerve injury at POD 14 (Fig. 3). CCR2 mRNA was no longer detected in lumbar DRGs taken from injured animals on POD35 (data not shown).

Activation of numerous chemokine receptors in addition to CCR2 might potentially be involved in the production of sensory neuron hyperexcitability and pain [3,4,26]. The chemokine SDF-1/CXCL12 and its receptor CXCR4 are constitutively expressed by peripheral nerves [3,27-29] and chemokines that activate CCR5 and CXCR3 receptors, such as RANTES and IP-10, are synthesized in association with neuroinflammatory responses [16,30]. To investigate whether chemokine receptors, in addition to CCR2, are upregulated following focal nerve demyelination, in situ hybridization studies were performed on injured rat DRG tissue sections.

**Figure 4:**
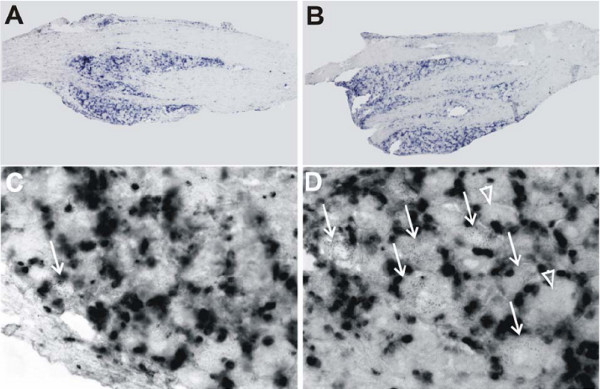
Expression of CXCR4 mRNA in rat lumbar DRG ipsilateral to focal nerve demyelination. Low (**A**) and high power (**C**) photomicrographs of CXCR4 mRNA transcripts present in lumbar DRG removed from vehicle-treated rodents at POD14 (n = 3). Many non-neuronal cells strongly expressed CXCR4. (**C**) Some presumptive neurons expressed low levels of CXCR4 mRNA (white arrow). Low (**B**) and high power (**D**) photomicrographs of CXCR4 mRNA transcripts present in lumbar DRGs derived from injured rats at POD14 (n = 4). The expression level and number of non-neuronal cells exhibiting CXCR4 mRNA transcripts in the lumbar DRG did not change following focal nerve demyelination. However, many neurons upregulated CXCR4 mRNA expression (**D**; white arrows indicate neurons with low levels of mRNA transcripts; white arrowhead points to a neuron lacking CXCR4 mRNA expression). (Scale bar is 1 mm (**A **and **B**); 40 μm (**C **and **D**).

Basal expression of CXCR4 mRNA was predominantly detected in non-neuronal cells of the lumbar DRG derived from naïve animals (data not shown). This level of non-neuronal expression did not change appreciably following vehicle treatment (Fig. 4A and 4C) or focal nerve demyelination at POD14 (Fig. 4B). Despite little change in non-neuronal cells, there was an increase in the number of neurons expressing CXCR4 mRNA transcripts by POD14 in the lumbar DRG ipsilateral to the nerve lesion (Fig. 4D). This pattern of staining was also observed in DRG contralateral to the nerve injury (data not shown). DRGs taken from injured animals on POD35 exhibited CXCR4 mRNA staining that was similar to that seen in naïve and vehicle-treated animals (data not shown).

**Figure 5:**
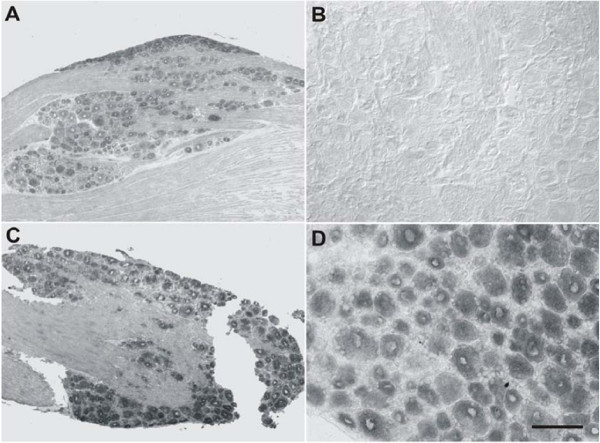
Expression of CXCR3 and CCR5 mRNA in rat lumbar DRG following focal nerve demyelination. (**A**) Many sensory neurons in the lumbar DRG removed from vehicle-treated rats exhibited CXCR3 mRNA transcripts at POD14 (n = 3). (**B**) CCR5 mRNA expression was absent from the lumbar DRG of vehicle-treated rats at POD14 (n = 3). (**C**) CXCR3 mRNA expression patterns in sensory neurons subjected to focal nerve demyelination did not differ from vehicle-treated rodents at POD14 (n = 4), but there was an increase in the intensity of CXCR3 mRNA expression. (**D**) Many neurons in the injured rat lumbar DRG expressed CCR5 transcripts at POD14 (n = 4). Scale bar is 250 μm (**A **and **C**); 100 μm (**B **and **D**).

Next we used in situ hybridization to examine the expression of the chemokine receptors CXCR3 and CCR5 in the DRG associated with the LPC-induced focal demyelination. These are the receptors for the chemokines IP-10 and RANTES (among other chemokines) respectively. Many sensory neurons were positive for CXCR3 mRNA in tissues taken from both naïve (data not shown) and sham-treated rodents at POD14 (Fig. 5A). The pattern of neuronal expression of CXCR3 mRNA at POD14 following focal nerve demyelination did not change relative to naïve or sham-treated tissue, but the nerve injury did increase the intensity of CXCR3 mRNA expression in the DRG ipsilateral to the injury (Fig. 5C), as well as in the contralateral DRG (data not shown).

Unlike CXCR3 expression, lumbar DRG in naïve (data not shown) and sham-treated rats at POD14 (Fig. 5B) were devoid of CCR5 mRNA transcripts. As with CCR2 expression in sensory neurons, CCR5 mRNA transcript levels were strongly increased in many sensory neurons ipsilateral and contralateral to the nerve injury at POD14 (Fig. 5D). Lumbar DRGs derived from injured animals on POD35 showed no CCR5 mRNA staining (data not shown).

### Chemokine expression in response to LPC-induced demyelination of the sciatic nerve

**Figure 6:**
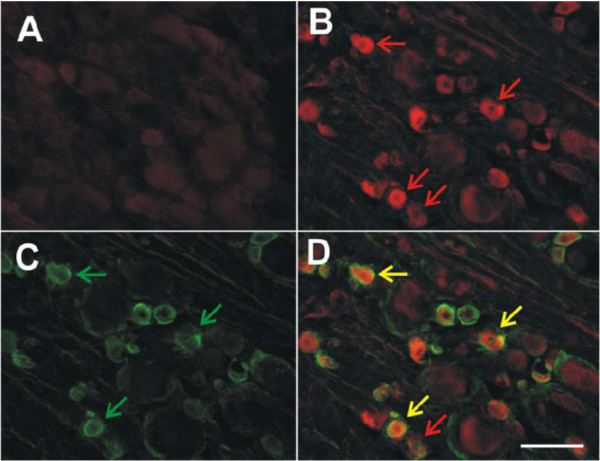
Colocalization of MCP-1 immunoreactivity (ir) and isolectin B_4_(IB_4_)-binding in the lumbar DRG ipsilateral to LPC-induced demyelination injury at POD7. IB_4_-binding in rat DRG neurons distinguishes C-fiber nociceptors. (**A**) Naïve rat lumbar DRG were completely negative for MCP-1ir. (**B**) Lumbar DRG ipsilateral to LPC-induced sciatic nerve injury exhibited numerous small diameter neurons that are MCP-1 positive at POD7 (red arrows). (**C**) Numerous small IB_4_-binding presumptive nociceptors are present in the same DRG tissue section (green arrows). (**D**) Merging panels B and C demonstrates the extent of colocalization present in lumbar DRG tissue section (yellow arrows). Note not all MCP-1ir neurons were positive for IB_4 _at POD7 (red arrow). Scale bar is 100 μm (**A**, **B**, **C **and **D**).

As the data discussed above indicates strong expression of several chemokine receptors by sensory neurons following LPC-induced demyelination injury, we sought to determine whether sensory neurons would also exhibit changes in the expression of chemokines that are possible ligands for these receptors under the same circumstances. Using immunocyctochemistry, we examined both the nerve lesion site and lumbar DRG associated with the injured nerve for MCP-1/CCL2 protein expression. Despite the multitude of ED-1-immunopositive macrophages in the injured sciatic nerve, MCP-1/CCL2-immunoreactive cells were absent from the nerve lesion site on POD1, 3, 7, 14 (data not shown), lumbar DRG removed from naïve rats (data not shown) and lumbar DRG from vehicle-treated rats at POD7 (Fig. 6A). In sharp contrast, numerous MCP-1 immunopositive neurons were present in associated lumbar ganglia of the LPC-treated rats by POD7 (Fig. 6B). The diameter of MCP-1 immunoreactive neurons in the LPC-treated animals averaged 26.49 ± 0.47 μm (n = 6). Interestingly, no MCP-1 protein immunoreactivity was detectable in injured DRG non-neuronal cells at any time point examined.

Once again, we co-stained DRG sections with IB_4_, a marker for C-fiber nociceptors. Many IB_4_-binding neurons were present in lumbar DRG of naïve, sham-operated and nerve-injured rats at POD7 and POD 14 (Fig. 6C) and Fig. 3, respectively). The average number of IB_4 _neurons present in the lumbar DRG was 47.87 ± 1.44% (n = 6 per condition) and did not significantly differ across treatment groups (sham and injured at POD14) (Fig. 3; p > 0.1). A series of MCP-1 immunopositive/IB4-binding colocalization experiments demonstrated that a significant portion of the MCP-1 positive neurons co-localized with IB_4 _at POD 7 and POD 14 (Fig. 6B, D; Fig. 3). More specifically, just over half of the MCP-1 immunoreactive neurons present at POD14 in DRG ipsilateral to nerve injury were also positive for IB_4 _(Fig. 3; 20.12 ± 0.75%; n = 6).

In a previous study, it was shown that in a rodent model of spinal stenosis, chronic compression of the DRG, there was increased MCP-1 expression and increased excitability of sensory neurons in injured and adjacent uninjured DRG [8]. Therefore, we wished to see whether MCP-1 activity was altered in the L4/L5 DRG contralateral to the nerve injury. Increased numbers of MCP-1-immunoreactive cells were present in ganglia contralateral to nerve injury (Fig. 3; 34.27 ± 1.17%; n = 6) and 13.32 ± 0.57% (n = 6) exhibited IB4 colocalization. Relatively few neurons were positive for MCP-1 in ganglia ipsi- or contralateral to the injury site in sham-treated animals (Fig. 3; <5%) and IB4 colocalization was rare (Fig. 3).

**Figure 7:**
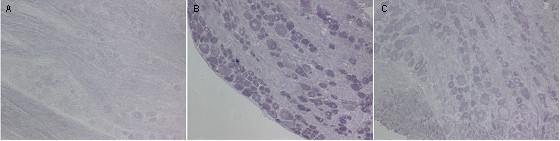
Expression of RANTES mRNA in rat lumbar DRG following focal nerve demyelination. (**A**) RANTES mRNA expression was absent from the lumbar DRG of vehicle-treated rats at POD14 (n = 3). (**B**) Many sensory neurons in the DRG ipsilateral to the nerve injury at POD 14 were positive for RANTES mRNA (**C**) Numerous sensory neurons in the DRG contralateral to the nerve injury displayed expression of RANTES mRNA, albeit at a lower level when compared with the DRG ipsilateral to the nerve injury. Scale bar is 250 μm (**A **and **C**); 100 μm (**B **and **D**).

As noted above, nearly every neuron in the DRG ipsilateral to the nerve injury upregulated CCR5. We used in situ hybridization to study the levels of RANTES expression, one known ligand for CCR5, in the DRG ipsilateral and contralateral to the nerve injury. RANTES expression was absent in the L4/L5 DRG in vehicle treated rats (Fig 7A). We found that after nerve injury, RANTES expression was strongly upregulated in DRG neurons ipsilateral and contralateral to the nerve injury, when compared to sham control tissue. Like CCR5, RANTES was expressed in small, medium and large sensory neurons (Fig 7B, C).

SDF-1 is the unique ligand for the CXCR4 receptor [31,32]. It has been reported that Schwann cells in the peripheral nerve express SDF-1 and that its expression increases moderately after nerve injury [3,29,33]. Using in situ hybridization we examined lumbar DRG associated with the injured sciatic nerve for SDF-1 expression at POD14. Many lumbar DRG non-neuronal cells present in both the naïve (data not shown) and vehicle-treated rats were positive for SDF-1 mRNA transcripts (Additional file 2A, C). Neither the pattern nor the expression levels of SDF-1 mRNA transcripts changed following focal nerve demyelination at POD14 (Additional file 2B, D).

**Figure 8:**
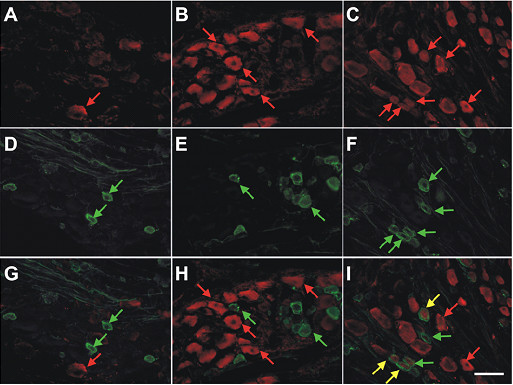
Colocalization of IP-10 immunoreactivity (-ir) and isolectin B4 (IB_4_)-binding neurons in the lumbar DRG of naïve rat and rats subjected to LPC-induced nerve injury. IB_4_-binding in rat DRG neurons distinguishes a population of C-fiber nociceptors. **A**) The majority of IP-10-ir cells were limited to medium diameter neurons in the lumbar DRG from vehicle-treated rats (red arrows). **D**) IB_4_-binding small diameter presumptive nociceptors (green arrows) did not colocalize with IP-10-ir lumbar DRG neurons from vehicle-treated rodents at POD7 (**G**, merged images). **B**) Lumbar DRG ipsilateral to focal nerve demyelination exhibited numerous medium and small diameter IP-10-ir neurons at POD7. Limited numbers of neurons were positive for both IP-10-ir (**B**, red arrows) and IB4-binding (**E**, green arrows) on POD7 (**H**, merged images). **C**) Many IP-10-ir neurons (red arrows) colocalized with IB_4_-binding neurons (**F**, green arrows) at POD14 (**I**, merged images). Yellow arrows indicate colocalized cells. Scale bar is 100 μm.

IP-10 is one of three ligands that bind to the CXCR3 receptor [34-36]. Little is known, however, concerning a role for IP-10 in sensory neuron function. We demonstrated that the sensory neurons in both vehicle-treated and injured lumbar DRGs exhibited appreciable levels of CXCR3 expression (Fig. 5A, C). Similarly, lumbar DRG from both naïve (data not shown) and vehicle-treated rodents displayed constitutive IP-10 immunoreactivity (12.04 ± 1.2%, at least 400 cells/DRG from each of 6 vehicle-treated animals) (Fig. 3, Fig. 8A), both ipsi- and contralateral to the site of nerve injury. The mean diameter of IP-10-immunopositive neurons in the vehicle-treated rat was 47.53 ± 1.7 μm. Seven days after LPC-induced focal nerve demyelination, the number of IP-10-immunopositive neurons in injured DRG had increased over two-fold to 30.41 ± 2.9% (n = 6) (Fig. 3, Fig. 8B). The average cell diameter of IP-10 immunoreactive neurons on POD7 was significantly reduced to 34.49 ± 0.98 μm. By POD14, the number of IP-10 immunoreactive sensory neurons had increased over three-fold (when compared to the vehicle-treated rat lumbar DRGs) to 36.8 ± 4.9% of the total neuronal population (Fig. 3, Fig. 8C), and the average neuronal diameter was further reduced to 29.74 ± 1.1 μm (n = 6).

A reduction in the average diameter of IP-10-immunopositive neurons concurrent with a three-fold increase in the number of neurons implies upregulated expression of IP-10 occurred in cells normally negative for the chemokine. Similar to previous findings in subpopulations of cells positive for the neurotrophin brain-derived neurotrophic factor (BDNF) by Obata and colleagues [37], this change in neuronal phenotype may be indicative of pathophysiological changes in primary afferent neurons following focal nerve demyelination. To determine the degree to which small, presumably nociceptive IB4-binding neurons, also displayed IP-10 immunoreactivity following LPC-induced nerve demyelination, we performed a series of colocalization experiments (Fig. 8D–I). Our analysis revealed that generally speaking, IP-10 immunoreactive neurons did not co-localize with IB4-binding neurons in vehicle-treated lumbar DRG (Fig. 3, Fig. 8D, G). On POD7, 9.39 ± 1.64% of the total neurons exhibited both IP-10 immunoreactivity and IB4-binding (n = 6) (Fig. 3, Fig. 8E, H). However, a two-fold increase in the number of neurons positive for both IP-10-immunoreactivity and IB4-binding (19.08 ± 2.31%; n = 6) was evident at POD14 (p < 0.01) (Fig. 3, Fig. 8F, I).

### Chemokines increase [Ca^2+^]_i _in DRG cells subjected to LPC-induced nerve demyelination

Activation of chemokine receptors expressed by primary sensory neurons results in excitation and in the increase in the intracellular Ca^2+^concentration [4]. To compliment the anatomical observations of upregulated chemokine receptor expression, we used fura-2 imaging of chemokine-induced increases in [Ca^2+^]i in acutely isolated rat DRG neurons as a measure of functional chemokine receptor expression. We acutely isolated the DRG cells from both the ipsilateral and contralateral sides of nerve injured animals and sham controls. For all experiments, the chemokines MCP-1, SDF-1, RANTES and IP-10 were added in random order to the cells, after which capsaicin, high K+(50 mM) and ATP were added to assess the cells' identity and viability, respectively. A response to high K+ stimulation indicates the presence of voltage dependent Ca2+ channels, which is indicative of neurons. Additionally, a positive response to capsaicin as well as high K+ indicates the cell is a nociceptor expressing the TRPV1 channel. A response to ATP, which activates purinergic receptors, without one to High K+ and/or capsaicin, indicates a non neuronal cell, presumably a type of glial cell such as a satellite glial cell or Schwann cell. The concentrations of chemokines used in these experiments were all supramaximal to ensure activation of any expressed receptors.

**Figure 9:**
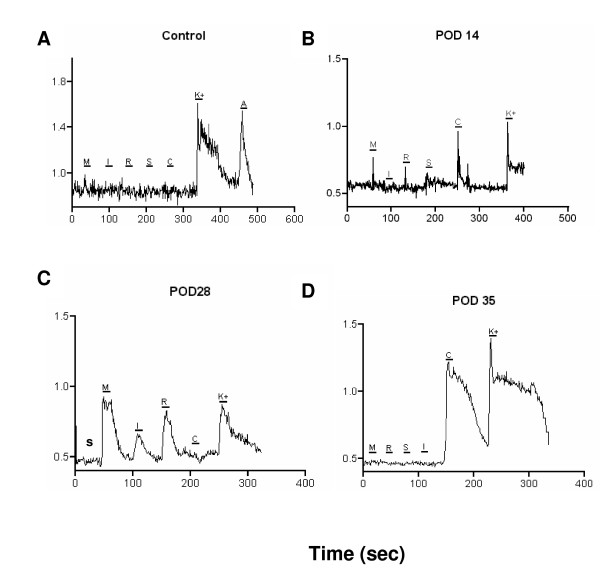
Chemokines increased [Ca2+]i levels in acutely isolated rat DRG cells following focal demyelination injury. The figure shows examples of responses of cells acutely isolated from rat DRGs ipsilateral to the nerve injury at various days after a focal demyelination injury. Under normal conditions, cells rarely respond to any chemokine but did respond to other stimuli such as high K or ATP (**A**). However, there was an increased responsiveness of the cells, the majority of which could be characterized as neurons, between post-operative days 14–28 (**B and C, respectively**). The frequency of the responses to chemokines returned to approximately the same level as control animals by post-operative day 35 (**D**). For all experiments, MCP-1 (**M**), IP-10 (**I**), RANTES (**R**), SDF1 (**S**) were applied at a concentration of 100 nM. Capsaicin (**C**), high K (**K**) and ATP (**A**) were applied at concentrations of 100 nM, 50 mM and 100 uM, respectively.

It was evident that an increased number of cells responded to MCP-1 application from POD 14–28 in the DRG cells ipsilateral to the nerve injury (Fig. 9B and 9C), (Table 1), when compared to vehicle-treated control animals (Fig 9A). The number of cells responding went from 5.8% to 31.7% by POD 28. The majority of these cells were characterized as neurons based on their positive responses to capsaicin and/or high K+ (Table 2). Many cells also exhibited increases in [Ca^2+^]i in response to the other chemokines tested (i.e. SDF1, RANTES and IP-10) and the frequency of these responses was always greatest by POD 28 in the nerve injured animals. Chemokine-induced changes in dissociated DRG were not limited to sensory neurons, but also included occasional non-neuronal cells (Table 2). It was also noted that at POD 14, most cells responded to only one chemokine. However, at POD 28, when upregulated chemokine signaling was at its greatest, most cells responded to multiple chemokines, indicating that single neurons expressed multiple chemokine receptors. In the DRG contralateral to the nerve injury, there was an upward trend in chemokine signaling at POD 14–28 (Table 1), but this did not reach significance. By POD 35, dissociated DRG cell responses to chemokines had returned to baseline levels (Fig. 9D, Table 1). Thus, the fura-2 imaging generally confirmed the observed upregulated expression and function of chemokine signaling in DRG cells subjected to a peripheral nerve demyelination.

**Table 1:** Table illustrates the chemokine response profile of acutely dissociated DRG cells from rats with and without peripheral nerve injury (n = 5/group). The cells were imaged while the various chemokines were added to the bathing solution. A rise in (Ca)i after chemokine application was indicative of the expression of a functional chemokine receptor. After chemokine application, capsaicin, high K+ and ATP were used to characterize the identity of the cells (see text). Note the upregulation of chemokine signaling at 14 and 28 days after peripheral nerve demyelination in isolated DRG cells ipsilateral to the nerve injury. There was also an upward trend in chemokine signaling in the cells isolated from the DRG contralateral to nerve injury at POD 14–28. By POD 35, chemokine responsiveness returned to baseline levels.

	**Control**		**POD 14**		**POD 28**		**POD 35**	
	
**Chemokine**	Ipsilateral	Contralateral	Ipsilateral	Contralateral	Ipsilateral	Contralateral	Ipsilateral	Contralateral
**MCP-1**	5.8%	7.9%	11.6%	2.5%	31.7% **	15.1%	2.4%	0.0%
**SDF1**	5.8%	7.0%	2.3%	5.0%	25% **	8.2%	7.3%	6.6%
**RANTES**	5.8%	7.0%	23.2% **	2.5%	8.3%	13.6%	9.7%	3.3%
**IP-10**	9.6%	8.8%	0.0%	20.0%	21.1%*	8.2%	7.3%	6.6%

*p < 0.01

** p < 0.05

n = 5/age group

**Table 2:** At 28 days, the majority of cells responded to capsaicin or high K+, indicating that most of the chemokine responsive cells were neurons.

**Capsaicin/High K+/ATP **positive (TRPV1 expressing nociceptor)	39.1%
**High K+/ATP **positive only (Non TRPV1-expressing neuron)	39.1%
**ATP **positive only (Non-neuronal cell)	21.7%

### CCR2 receptor antagonist attenuates bilateral focal nerve demyelination induced tactile hyperalgesia

**Figure 10:**
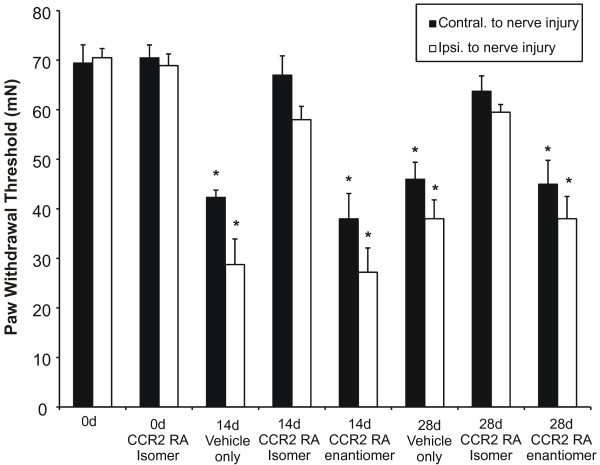
CCR2 receptor antagonist (CCR2 RA-[**R**]) administration reversed existing nociceptive behavior. Animals were subjected to a nerve demyelination injury on day 0 and nociceptive behavior was assessed for 28 days. On days 14 and 28 post-surgery, animals received 5 mg/kg CCR2 RA-[**R**] or 5 mg/kg of its inactive enantiomer, (CCR2 RA-[**S**], or saline by intraperitoneal injection, and behavioral responses were tested 1 h later. Administration of the CCR2 RA-[**R**] to focal nerve demyelination injured rats resulted in a significant bilateral increase of mN force required to elicit a paw withdrawal compared with vehicle-treated controls and animals subjected to CCR2 RA-[**S**]. Nociceptive behavior in vehicle-treated controls and animals subjected to CCR2 RA-[**S**] differed significantly from day 0 pre-injury baseline responses (*p < 0.01). Data represent means ± SE.

Using LPC nerve-injured animals, we tested the effect of a single i.p injection of a CCR2 receptor antagonist (CCR2 RA-**[R]**) or its inactive enantiomer (CCR2 RA-**[S]**) on nociceptive behavior at POD14 and POD28 (10 mg/kg). Neither vehicle nor CCR2 RA-**[S] **administration at day 14 or day 28 had an effect on the bilateral mechanical PWT when tested 1 hour later (Fig. 10). In contrast, bilateral increases in PWT were observed one hour after administration of CCR2 RA-**[R]**. These PWTs did not differ from pre-operative basal threshold levels (Fig. 10). The effects of CCR2 RA-**[R] **were stereospecific as administration of an equal dose of the inactive stereoisomer did not inhibit pain behavior. All PWTs returned to pre-drug administration levels within 24 hours (data not shown).

## Discussion

Previous work carried out in our own and other laboratories has indicated that chemokine signaling may contribute to the genesis and maintenance of neuropathic pain [11,17,18]. Thus, the present studies were designed to investigate a potential association between focal nerve demyelination, neuropathic pain behavior and chemokine signaling in DRG neurons. Based on our previous studies, we hypothesized that the focal demyelination of the sciatic nerve, a known rodent model of neuropathic pain [12], would result in upregulated chemokine expression and chemokine receptor signaling in DRG neurons. Indeed, we observed the predicted increases for several chemokines and their receptors. Importantly, as the PWT decreased over 14 days post-injury, chemokines, chemokine receptors and chemokine/receptor signaling increased significantly. This occurred not only in the DRG directly associated with the injured nerve, but also to a lesser degree, in the DRG directly contralateral to the nerve injury. Bilateral nociceptive behavior was apparent between days 3–28 and could be stereospecifically attenuated with a CCR2 receptor antagonist on days 14 and 28. Moreover, five weeks following injury, both the neuropathic pain behavior and the incidence of chemokine signaling were greatly diminished. Together with the known cellular effects produced by chemokines on sensory neurons [3,7], these results suggest that the changes in sensory neuron chemokine/receptor signaling may be central to the maintenance phase of neuropathic pain behavior in particular.

The results of our previous studies, together with the present results on LPC-associated neuropathy, point to a significant role for chemokine signaling expressed directly by peripheral nerves. For example, we have previously demonstrated that activation of chemokine receptors expressed by cultured or acutely isolated DRG neurons produces increased (Ca^2+^)_i_, or neural excitation [4,38]. Indeed, following upregulation of CCR2 by sensory neurons in whole DRG derived from animals exhibiting neuropathic pain, application of MCP-1 produces powerful excitation [8]. The mechanism underlying this response probably involves activation of phospholipase C-induced degradation of PIP2, production and concomitant transactivation of TRPV1 and/or TRPA1 together with inhibition of K+ conductance [9,10].

The experiments reported here suggest a model in which focal nerve demyelination produces a concomitant upregulation of several chemokines and their receptors in the cell bodies of sensory neurons in the DRG. We have observed that chemokines expressed by DRG neurons, including MCP1, IP10 and SDF1, can be packaged into secretory vesicles and released upon depolarization [9]. Presumably, chemokines released in this fashion may influence neural cells in the local vicinity eliciting excitation as described above. Such activation would produce further chemokine release and excitation driving the overall excitability of the chemokine sensitive neurons to new levels. The resulting neuronal behavior may explain certain aspects of pathologically maintained neuronal states of depolarization or electrical hyperexcitability of peripheral sensory neurons [39-41]. In addition to the chronic maintenance of sensory neuron hyperexcitability, release of chemokines such as MCP-1 and fracktalkine from central axon terminals in the spinal cord may initiate microglial-mediated neuropathic pain states [7,11,42-44]. However, it is important to note that pharmacological therapies which inhibit microglial activation and effectively attenuate the development of hyperalgesia and allodynia have no effects on preexisting nociceptive pain behavior [45].

As we have demonstrated, the exact pattern of changes in chemokine signaling observed following focal nerve demyelination depends on the particular chemokine receptor and ligand examined. There are over 50 known chemokines and 20 chemokine receptors [32], and it is obviously not feasible to study all of these simultaneously. However, the receptors studied in the present experiments represent obvious candidates for a role in peripheral neuropathy. Chemokines that signal via the CCR2, CCR5, CXCR3 and CXCR4 receptors have previously been shown to influence the behavior of sensory neurons [3,4,6,8,11,17]. Furthermore, many of these receptors can be upregulated in leukocytes by mechanisms suggesting that regulation of their expression may often be coordinated through the same transcriptional control mechanisms [46].

The four chemokines/chemokine receptors that we studied all displayed different patterns of expression in response to focal nerve demyelination, suggesting different roles in the genesis of pain or other functions in the DRG. The upregulation of MCP-1 and the CCR2 chemokine receptor observed in association with focal nerve demyelination is similar to the pattern we previously observed using a spinal stenosis model of neuropathic pain [8]. Indeed, CCR2 receptor deficient mice are resistant to the induction of some sensory neuropathies, highlighting the potential importance of this chemokine signaling system [11]. In the current experiments, we utilized a Ca^2+ ^imaging paradigm in lieu of electrophysiological recording, as chemokine-induced increased neuronal excitability would be expected to be correlated with a chemokine induced increase in (Ca^2+^)_I_. The observed increase and subsequent decrease in MCP-1-induced Ca^2+^responsiveness in acutely isolated DRG neurons over time generally correlated with the anatomical observations of receptor expression, and both effects returned to baseline by POD 35. Importantly, the ability of the CCR2 receptor antagonist to attenuate bilateral nociceptive behavior at both 14 and 28 days after nerve injury strongly suggests an integral role for MCP-1/CCR2 signaling in maintaining this phase of pain hypersensitivity.

Although the CCR2 antagonist was effective in blocking pain hypersensitivity, more than one chemokine or chemokine receptor was upregulated in this neuropathic pain model. The particular effectiveness of CCR2 block could be due to the fact that upregulation of chemokines can be bilaterally expressed in different populations of sensory neurons following nerve injury, as is this case of cholecystokinin vasoactive inhibitory peptide and neuropeptide Y [43,47]. In our experiments over 50% of the cells that upregulated MCP-1 also expressed IB-4, which is a marker for C-fiber nociceptors that are responsible for transmitting pain information. This differs from the case of IP-10, where a majority of the neurons upregulating this chemokine did not co-localize with IB-4. As such, it is possible that the population of neurons that upregulates CCR2 signaling is particularly linked to the production of neuronal hyperexcitability. It is also likely that the CCR2R antagonist may impact non-neuronal cells within the CNS, such as microglial cells, which are known to express CCR2 in the spinal cord and contribute to the development of chronic pain states [11,48,49].

The precise location of action of the CCR2 antagonist is not known. However, it has been shown that the blood nerve barrier is less restrictive than the blood brain barrier [50], with the cell body rich area of the DRG being vulnerable to extravascular leakage. Given these studies, it is likely that the CCR2 R antagonist reached the cell bodies of the DRG. As activation of CCR2 receptors in the DRG is probably of considerable importance in the production of pain behavior it is likely that block of these receptors contributes to the antinociceptive effects of the CCR2 antagonist.

In the face of the effectiveness of CCR2 receptor block either pharmacologically (Fig 9) or genetically [11], the function of other types of upregulated chemokine signaling to chronic pain behavior is not immediately obvious. Like CCR2, the CCR5 receptor function and its ligand, RANTES, were also strongly upregulated in DRG neurons in response to focal demyelination. It has previously been shown that RANTES may be important in other chronic pain situations [51]. Our findings in this sciatic nerve injury model differ from a report by Taskinen and Royotta [16] which demonstrated bilateral upregulation of non-neuronal RANTES for up to four weeks following sciatic nerve transection in the rat. The apparent differences may be due to the nature of the nerve injuries. Alternatively, CCR5 may also be activated by a number of other chemokine ligands which we did not measure [52]. Chemokine interactions with CCR5 may also depress the analgesic action of endogenous opioids and/or sensitize TRPV1 [10,53,54] thereby generally promoting hyperalgesia.

The signaling pattern of IP-10 and CXCR3 receptors in the DRG differs in some respects as there is appreciable basal neuronal expression of both CXCR3 receptors and IP-10. In spite of this, few Ca^2+ ^neuronal responses were observed in the naïve or sham animals, perhaps because of desensitization resulting from ongoing receptor activation induced by constitutive expression of IP-10. Neuronal expression of IP-10/CXCR3 under basal conditions may have a specific role to play that is analogous to the expression and release of fractalkine by neurons [42,55]. Following strong neuronal excitation in the peripheral nervous system (i.e. trauma or disease), IP-10 may be released within the DRG and/or from central terminals in the spinal cord dorsal horn resulting in both local and distant glial activation [26,56,57].

Focal nerve demyelination changes in SDF-1 signaling via the CXCR4 receptor show still another pattern. In this case, the chemokine receptor is not generally expressed in neurons, but in satellite glia and Schwann cells. Upregulated CXCR4 expression, however, is primarily restricted to neurons making them a potential target for the release of SDF-1 from glia. It is interesting to note that a role for Schwann cell release of SDF-1 and for neuronally-expressed CXCR4 receptors has also been suggested in recently proposed models of HIV-1 and NRTI related neuropathies [14,51].

It is clear that focal nerve demyelination injury-induced behavioral changes are correlated with widespread changes in the neuronal expression of chemokine/receptors and that the pattern of expression of each chemokine and its receptor is unique, suggesting that the influence of chemokine signaling on rodent nociceptive behavior may be complex. It should also be noted that the upregulated expression of different chemokines and receptors that we have observed may occur as part of a cytokine "cascade", where the expression of one chemokine or its receptor is dependent on previous events. If that is the case it is also possible that drugs which block several upregulated chemokine receptors may also prove to be effective if they are upstream of CCR2 expression.

In the course of these studies, we also noted that the PWT to mechanical stimulation decreased bilaterally. The degree of threshold change in the hindpaw contralateral to the nerve injury was qualitatively similar but smaller in magnitude, and briefer in time course, when compared with the hindpaw ipsilateral to the lesion. This type of bilateral hyperalgesia has previously been described in other rodent models of neuropathic pain [58-67]. As such, it is of interest to compare the LPC-induced peripheral nerve pain model with previously described rodent inflammatory pain models which demonstrated bilateral tactile pain behavior [60] and contralateral changes in the DRG [65,68]. Milligan et al. [69] suggested that this phenomenon is likely due to changes in the spinal dorsal horn. This type of spinal mechanism may drive both bilatateral pain sensitivity and contralateral DRG changes in chemokines/receptors following unilateral sciatic nerve demyelination by releasing cytokines from activated microglia in the spinal cord dorsal horn following chronic activity in injured DRG afferent neurons [70]. Spinal cord-derived cytokines or growth factors such as TNF-α, IL-1β, IL-6 and/or BDNF, may also directly signal contralateral lumbar DRG neurons through receptors present on primary afferent central terminations [71,72]. Perhaps not surprisingly, blockade of the action of TNF-α or inhibition of glial metabolic activity can attenuate bilateral nociceptive pain behavior [69,73], while the low dose administration of a gap junction protein decoupler only extinguishes only contralateral pain behavior [74]. Although current trends in pain research favor bilateral spinal cord glial activation as a mechanism of central activation in the spinal cord, it does not appear to be central to all pain conditions [61]. It is also interesting to note in the context of the present set of investigations that TNF-α can upregulate MCP-1 expression by sensory neurons [75], further supporting the possibility that it may function as an upstream regulator of chemokine signaling in the DRG.

Alternatively, aspects of bilateral tactile hyperalgesia may be due to spontaneous ectopic activity in A, but not C-fibers. This type of ongoing ectopic hyperexcitability in primary sensory neurons post-injury can occur in both injured neurons and adjacent, uninjured neurons [20,37,76,77]. Changes necessary for this type of mechanism implicate modification of the electrical properties of the neurons [78-80]. Nerve demyelination in the mid-thigh may effectively trigger just this type of change in the primary sensory neuron (i.e. neurochemistry and physiology of primary afferent neurons), which then contribute to central sensitization and higher levels of nociceptive sensory processing. Taken together, the evidence of chronic changes in chemokine/receptor protein expression and the ability of certain chemokines to excite neuronal subpopulations [6,8] is suggestive of a potential scenario.

Another behavioral component commonly observed with rodent pain models, especially those accompanied by robust inflammatory responses, is the presence of thermal hyperalgesia. Despite a presence of thermal hyperalgesia in the mouse focal nerve demyelination model [12], the rat nerve demyelination model does not exhibit changes in response to temperature. Thermal hyperalgesia is largely thought to be a pain-related symptom caused by peripheral sensitization. The absence of thermal hyperalgesia would suggest that there is a lack of ongoing inflammatory mediator-initiated sensory neuron signaling. Lack of thermal hyperalgesia in peripheral nerve injury models of pain, although not common, include perineural gp-120 administration [81]; Bhangoo and White, unpublished observations) acidic saline-induced hyperalgesia [63] and chronic constriction injury performed in a 5-HT transporter knockout mouse [82]. Lack of thermal hyperalgesia in the model of muscle pain and CCI implicate central descending mechanisms for the display of bilateral hyperalgesia. It is possible that similar mechanisms are operating within the rat nerve demyelination injury model.

Taken together the data suggest that upregulation of chemokine signaling by sensory neurons may help to integrate several phenomena that account for changes in the properties of peripheral nerves resulting in bilateral pain hypersensitivity. The present results, together with previous studies [3], suggest that the mode of injury may determine which particular chemokines play a central role in maintaining the neuropathic pain state. Chemokine receptors may then represent novel targets for therapeutic intervention in demyelination associated neuropathic pain as well as other chronic pain states.

## Supplementary Material

Additional file 1 The chemical structures and full names of the CCR2 antagonist (CCR2-[**R**]) and its inactive enantiomer (CCR2-[**S**]). Click here for file

Additional file 2 Expression of SDF1 mRNA in rat lumbar DRG ipsilateral to focal nerve demyelination. (**A**) Many cells, both satellite glia and neurons, in the lumbar DRG removed from vehicle-treated rats exhibited SDF1 mRNA transcripts at POD14 (n = 3). (**B**) SDF1 mRNA expression did not change significantly in the lumbar DRG of LPC-treated rats at POD14 (n = 3). (**C**) A magnified photomicrograph of the lumbar DRG from a vehicle treated rat. (**D**) A magnified photomicrograph of the lumbar DRG removed from a LPC-treated rat at POD14. Click here for file
