# Supplementary material for: Delayed functional expression of neuronal chemokine receptors following focal nerve demyelination in the rat: a mechanism for the development of chronic sensitization of peripheral nociceptors
Source: Mol Pain. 2007 Dec 12;3:38. doi: 10.1186/1744-8069-3-38 (PMC2228278; doi:10.1186/1744-8069-3-38)
Supplement: Additional file 1 — The chemical structures and full names of the CCR2 antagonist (CCR2-[R]) and its inactive enantiomer (CCR2-[S]). [file 1744-8069-3-38-S1.doc]

**(R)**-4-Acetyl-1-(4-chloro-2-fluorophenyl)-5-cyclohexyl-3-hydroxy-1,5-dihydro-2H-pyrrol-2-one

**(S)**-4-Acetyl-1-(4-chloro-2-fluorophenyl)-5-cyclohexyl-3-hydroxy-1,5-dihydro-2H-pyrrol-2-one

[**R**] – active enantiomer

[**S**] – inactive enantiomer
