# Supplementary figures and images for: Delayed functional expression of neuronal chemokine receptors following focal nerve demyelination in the rat: a mechanism for the development of chronic sensitization of peripheral nociceptors
Source: Mol Pain. 2007 Dec 12;3:38. doi: 10.1186/1744-8069-3-38 (PMC2228278; doi:10.1186/1744-8069-3-38)

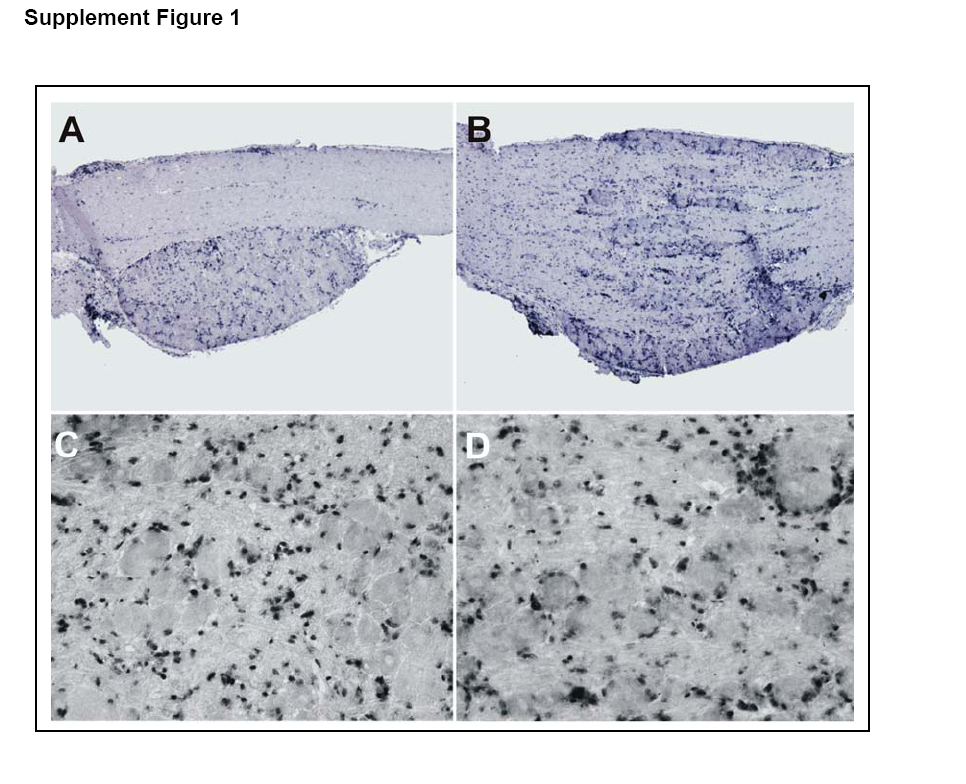

Supplement: Additional file 2 — Expression of SDF1 mRNA in rat lumbar DRG ipsilateral to focal nerve demyelination. (A) Many cells, both satellite glia and neurons, in the lumbar DRG removed from vehicle-treated rats exhibited SDF1 mRNA transcripts at POD14 (n = 3). (B) SDF1 mRNA expression did not change significantly in the lumbar DRG of LPC-treated rats at POD14 (n = 3). (C) A magnified photomicrograph of the lumbar DRG from a vehicle treated rat. (D) A magnified photomicrograph of the lumbar DRG removed from a LPC-treated rat at POD14. [file 1744-8069-3-38-S2.doc]
